# Soft Tissue Management Around the Dental Implant: A Comprehensive Review

**DOI:** 10.7759/cureus.48042

**Published:** 2023-10-31

**Authors:** Elizabeth P Jose, Priyanka Paul, Amit Reche

**Affiliations:** 1 Public Health Dentistry, Sharad Pawar Dental College, Datta Meghe Institute of Higher Education and Research, Wardha, IND

**Keywords:** dental implant, soft tissue management, peri-implantitis, peri-implant mucositis, osseointegration, augmentation, implant

## Abstract

In the modern world, there is an increasing concern among people regarding dental esthetics. Edentulism can impact one's appearance, affect the regular bite, and can even affect mental well-being. There are various options to replace the missing teeth, such as removable dentures, fixed crown and bridge prostheses, and resin-retained bridges. Various factors are evaluated before giving a suitable prosthesis for missing teeth. Implant installation is highly desired by patients as it has a high success and long-term survival rate when used to replace lost teeth. However, several difficulties relating to errors in treatment planning, surgery, soft tissue, and hard tissue care, and infections may compromise the efficacy of implant therapy. An increasing body of research indicates that long-term clinical stability and esthetics may be significantly impacted by the stability of the soft tissues around osseointegrated dental implants. Consequently, when implant therapy is planned, the dental surgeon has to have the necessary expertise to appropriately handle any possible causes of difficulties in addition to being able to carry out the necessary actions to maintain or develop stable soft tissue. Various augmentation procedures can be done for the correction of any deformity or inadequacy of soft tissues. Osseointegration is a fundamental part of the success of the implant treatment. It is the formation of a biological and functional connection between the bone and the implant increasing the stability of implant prosthesis. After the treatment, the patient should be counseled for regular and proper oral hygiene practices suitable for the implant. A proper follow-up has to be done after implant treatment in regular intervals. Any postoperative soft tissue complications, such as peri-implantitis or peri-implant mucositis, should be addressed immediately, and appropriate treatment has to be given. This article reviews about the procedures before and after the implant placement to prevent or treat soft tissue complications, ultimately leading to the success of the implant.

## Introduction and background

Oral implantology is of greatest interest in modern dentistry. A dental implant is considered an excellent option for the rehabilitation of missing teeth. It has a major advantage over conventional alternatives, as it offers greater stability and retention. In individuals who have compromised supporting bone or mucosa, dry mouth, allergies to denture materials, a severe gag reflex, vulnerability to candidiasis, diseases affecting craniofacial motor control, or in patients who demand the best bite force, esthetics, and function, endosseous dental implants may be preferable over conventional dentures [1]. However, since the implant derives support from the bone and the soft tissue around it, the possibilities of failure are also undeniable. For the success of the treatment, several factors should be taken into account, such as patient selection, implant loading, tissue management, and regular follow-up [2].

There should be an ample amount of soft and hard tissue with minimal occlusion for the success of the treatment. According to recent research, the stability of the soft tissues around osseointegrated dental implants may significantly affect the long-term clinical stability and esthetics of the soft tissues. Because of this, when implant therapy is planned, the clinician must not only be able to carry out the necessary actions to maintain or establish a stable soft tissue but also be aware of the potential sources for future complications and have the necessary expertise for their proper care [3]. Proper reconstruction and management of soft tissue along with osseointegration results in good esthetics. A functional implant should have a part that transverses the oral mucosa. Thus, it creates a biological connection with the living tissues. This connection has to be created during the healing process after the placement of an implant. Biologic differences are evident due to the intrusion of the foreign body (a component of the implant). It must be corrected using the proper surgical techniques and biomaterials design. The soft tissue barrier thus created is meant to shelter the underlying osseous structures [4]. For better stabilization of the implant, an adequate amount of keratinized gingiva is also mandatory. Therefore, the purpose of the review was to conduct a systematic literature review to analyze the ways of enhancing soft tissue health around the implant site and basic knowledge about possible complications and treatment.

## Review

Methodology

**Figure 1:**
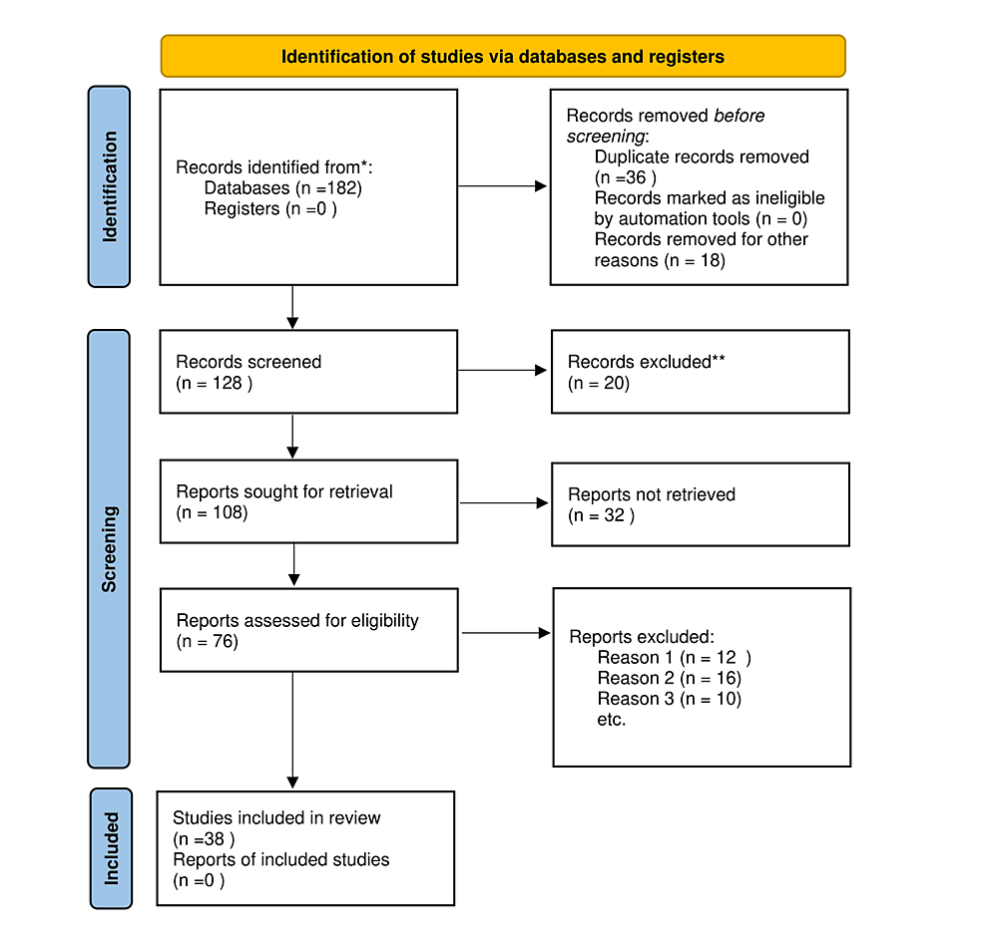
Selection process for articles included in this study Adopted from the Preferred Reporting Items for Systematic Reviews and Meta-Analyses (PRISMA).

We initiated a comprehensive search through PubMed and Google Scholar in October 2022 using keywords such as "peri-implantitis," "peri-implant mucositis," "osseointegration," "augmentation," "implant," (peri-implantitis[Title/Abstract]) OR ("peri-implantitis"[MeSH Terms]), ("peri-implant mucositis"[Title/Abstract]) OR ("peri-implant mucositis"[MeSH Terms]), ("osseointegration"[Title/Abstract]) OR ("osseointegration"[MeSH Terms]), ("augmentation"[Title/Abstract]) OR ("augmentation"[MeSH Terms]), ("implant"[Title/Abstract]) OR ("implant"[MeSH Terms]). A total of 38 articles were included. Figure 1 shows the Preferred Reporting Items for Systematic Reviews and Meta-Analyses (PRISMA) flow diagram for the search strategy.

Case selection and planning of treatment

The main aim of the treatment is to achieve osseointegration and the overall supportive anatomy of the implant site for the prosthesis. The patient has to be physically fit to receive the implant regarding the systemic health of the patient, the condition of the bone, and local factors around the site. Since the rate of resorption of crestal bone is higher in young individuals, it is advised to go for implant treatment after achieving complete maturation of the facial dentoskeletal structure. Smoking is contra-indicated in implant receivers since it interferes with the healing and successive osseointegration. Even though osteoporosis causes bone fragility and resorption, it is not a complete contraindication for implants. Implant placement is not desirable in patients who are in cytotoxic chemotherapy. It is advisable to seek an opinion from the patient's consulting physician who is on systemic drugs before the case planning. Oral lichen planus patients and people at high cancer risk have a lower chance of success, hence alternatives for implant treatment are recommended [5]. For the assessment and selection, a proper radiographic evaluation is necessary. The peri-oral radiograph gives an idea about the bone structure and local pathologies. There should be an adequate length and density for the residual bone otherwise various bone augmentation procedures could be planned. The diagnostic cast is mounted and planning is done after the radiographic examination [5,6]. The marginal bone loss shown by the radiological examination has been the primary parameter used to evaluate the effectiveness of oral implants [7-9]. The cross-sectional views of the dental arch that the imaging objectives provide to the clinician help visualize the spatial placement of the maxilla and mandible's anatomic frameworks, the quantity and quality of the available bone, the presence of intra-bony lesions, the occlusal arrangement, the number and size of implants, and the prosthesis layout. All of these details are critical for the effective execution of implant treatment and the assessment of the continuous efficiency of the implants [10]. In the field of implantology, a variety of radiographic methods are employed, including computed tomography (CT), orthopantomography (OPG), occlusal radiography, intraoral periapical radiography (IOPAR), conventional tomography, and cone-beam CT (CBCT). Most of the time, the practicing clinician makes the decision on which modality best meets their needs [11-13]. The American Academy of Oral and Maxillofacial Radiology (AAOMR) released a position paper on the application of radiology in dental implantology. The AAOMR recommended that cross-sectional imaging be used for the evaluation of all dental implant sites and that CBCT is currently the preferred imaging method to obtain such diagnostic data [14].

Soft tissue considerations

Healthy soft tissue around the implant site not only results in the success of the treatment but also helps in the esthetic finish of the treatment. The mucosa around the implant must surround the neck of the prosthesis to provide function and esthetics. An average of 3-4 mm thickness of mucosa is ideal. The reduced thickness of mucosa will result in the resorption of bone and lead to angular defects. There must be a crown portion component of mucosa in a range of 2-2.2 mm thickness and an apical part of 1.1-1.7 mm thickness. Between the teeth, there must be a vertical component of thickness ranging from 1 to 1.5 mm, which is essential around the implant [15]. For better stability of prosthesis, there should be adequate thickness of keratinized attached mucosa [4]. An average of 2 mm of keratinized mucosa and 1 mm of attached gingiva in the implant site is essential [16]. The reduced thickness of mucosa will result in the resorption of bone and lead to angular defects [15]. This helps in minimizing plaque accumulation, and soft tissue recession also limiting the peri-implant mucositis incidence. Ridge augmentation procedures are to be done prior to the implant placement if there is any ridge resorption. The blood supply to the implant site is not the same as to that of teeth since there is a lack of the periodontal ligament in the junction of the implant and bone. The complete vascular supply is provided by the supra periosteal vessels from the osseointegrated bones [4]. The soft tissue correction treatment before placing the implant is conventional methods such as augmentation procedures and graft techniques. Common augmentation procedures are mainly done prior to the implant placement. If there is a high frenal attachment, it should be relieved to avoid tissue tension after the surgery.

Augmentation procedures

Augmentation of soft and hard tissue in the implant site is done to obtain the desired volume around the implant site. These procedures can be done before, during the implant loading, or after the implant placement (Table 1) [16].

**Table 1:** Augmentation procedures Source: [16].

Before the implant placement	During the implant loading	After implant placement
Perform all necessary investigations. Conduct frenectomy if required. Utilize apical positioned flaps (APF) to increase keratinized mucosal thickness and deepen the vestibular sulcus. Apply a free gingival graft. Follow up with another APF to promote faster healing of the extraction socket.	Soft tissue correction goals during implant placement: Adjust vertical height and mucosal thickness. Techniques used: Free gingival grafts or subepithelial connective grafts. Benefits of soft tissue correction: (a) Reduces the number of surgeries for the patient. (b) Improves healing times. (c) Enhances overall patient satisfaction.	Second stage procedures: (a) Utilize a connective tissue graft (CTG). (b) Perform periosteum lifting, preferably using a split-thickness graft. (c) Aim to limit bone resorption. Maxilla and mandible procedures: (a) In both the maxilla and mandible, use apically positioned partial thickness flaps. (b) Note that the mandible lacks keratinized tissue, so a free gingival graft is applied. Esthetic zone considerations: (a) In the esthetic zone, employ the split-finger technique. (b) Combine it with the Palacci flap to create papilla. Osseointegrated implants: (a) Soft tissue augmentation is possible with osseointegrated implants. (b) Achieve this with a coronally positioned flap coupled with a connective tissue graft for excellent results.

Osseointegration

Osseointegration is an event in which there is the development of biological and functional relations between the implant and the vital bone structure around it. If this connection gets established, then there will be no relative motion between the two structures. Once the osseointegration is established, it is understood that the implant is biologically compatible with the adjacent tissues. It will result in the least possibility of systemic or localized irritation [17]. The healing of the bone and osseointegration is processed through a series of events. The placement and the cementless fixing of the implant operate through the formation of the adjacent mesenchymal cells and the development of a hematoma. These events are followed by the formation of woven and lamellar bone. The woven bone is formed intra-membranously and the lamellar bone is inside the spicules of the woven bone. These steps of osseointegration are coordinated by the blood cells at the bone-implant junction. They form the growth and differentiation factors to mediate the process. The osseointegration is confirmed by the appearance of marrow spaces. The marrow spaces must contain healthy osseous cells such as osteoclast, osteoblast, and osteocyte with mesenchymal cells with neovascularization [18]. This way, osseointegration is gradually attained by the inflammatory, formation, and re-modeling phase of the bone. However, if the peri-implant mucosa is not healthy enough, the healing and osseointegration are adversely affected.

Postoperative soft tissue complications and their management

The failure of a dental implant is frequently linked to the failure in osseointegration. If a dental implant is misplaced, moves, or exhibits loss of bone around the implant more than 1.0 mm in the first year and more than 0.2 mm in the following year, it is deemed a failure. Loss of bone surrounding the implant and ultimately implant loss may be the outcome of peri-implantitis [19]. A regular follow-up is needed for the assessment of tissue health around the implant. The possible complications around the implant are peri-implant peripheral giant cell granuloma, pyogenic granuloma, squamous cell carcinoma, metastatic carcinomas, malignant melanoma, etc. The more prevalent complications following the implant placement are peri-implantitis and peri-implant mucositis [20]. Peri-implant mucositis and peri-implantitis are two conditions that fall within the definition of peri-implant disease. A reversible inflammatory response in the soft tissues around an implant is referred to as peri-implant mucositis [21]. Peri-implantitis is an inflammatory response with bone loss in the surrounding tissues of an implant [22]. Basically, these are inflammatory conditions leading to the complications of peri-implant tissues. It is detected by bleeding or even exudate while probing, increased depth of probing, and the failure of osseointegration. Also, inflammation can be detected through various inflammatory biomarkers [23]. The most significant factor in dental implants failing is bacterial infections. It has been shown that the bacterial flora linked to peri-implantitis and periodontal diseases are almost identical [24].

The causes of infection can be cement excess, plaque accumulation, infections, or even can be due to the overload of the occlusal stresses of the prosthesis wearer. The main cause for peri-implant mucositis is plaque and it can be reverted. So, mucositis complications due to plaque accumulation can be prevented by regular oral hygiene practices, proper brushing, and flossing after meals [25]. The oral microbiota appears to be a determining factor in whether a dental implant is successful or unsuccessful. When an implant is introduced to the oral cavity, salivary pellicle, a protein coating, and oral microbes instantly cover it, colonizing it to form a microbial biofilm. Instead of specific scientific results, the therapeutic approaches suggested for treating peri-implant illnesses seem to be mostly based on either the data currently available for treating periodontitis or on clinical empirical values. An investigation conducted by Schwarz et al. [26] showed that antiseptic (0.2% chlorhexidine) therapy in conjunction with mechanical debridement with plastic curettes to treat peri-implant infection may result in analytically notable improvements in clinical attachment level, peri-implant probing pocket depth and bleeding on probing at six months compared with baseline. The fundamental component of treating periodontitis and peri-implantitis is surface debridement [27]. But for complicated cases, flap surgeries, application of nanocrystalline hydroxyapatite (NHA), and other surgical treatments are also done [28]. Instruments that are softer than titanium should be used to clean the dental implant, such as plastic scale tools, floss, interdental brushes, or polishing with a rubber cup and paste. Unlike metal and ultrasonic scalers, it has been demonstrated that they do not cause the surface of implants to become rough [29]. Sometimes even after proper hygiene practices, infection happens. Plaque buildup and bacterial biofilm development may be made easier by the screw-shaped nature of the implants and different titanium surface changes [30]. On such surfaces, mechanical debridement may only have a limited impact, and it is unlikely to completely eradicate all clinging microbes. To deal with bone defects in advanced peri-implantitis cases, regenerative approaches involving a membrane and a bone graft replacement have been proposed [27]. In these cases, the early treatment gives the best result. In patients without additional infections who have localized peri-implant issues, local medication delivery devices may be a viable therapy option. A prolonged high dosage of the antibacterial agent can be applied locally for many days by inserting tetracycline fibers into the afflicted location for 10 days. The long-term existence of peri-implant mucositis can end up in peri-implantitis. Peri-implantitis affects osseous tissue around the implant and is even more severe. Whereas the mucositis only affects the mucosa [31]. So, prevention of these conditions by regular follow-up and professional and self-cleaning of the implant is advised. Peri-implant mucositis can be rectified non-surgically and it has a good prognosis. However, peri-implantitis can only treated by means of surgery. Also, it has a poor prognosis [25]. Surfaces and mechanical debridement may only have a limited impact, and it is unlikely to completely eradicate all clinging microbes [32].

Surgical techniques

After surgical therapy, defects repaired with membrane-covered autogenous bone demonstrated much greater quantities of bone regrowth and re-osseointegration compared to those treated using the other four techniques: (1) membrane-covered autogenous graft, (2) autogenous bone grafts only, (3) membranes alone, and (4) a control access flap technique [33]. However, following such treatments, membrane exposure is a common consequence. When permeable expanded polytetrafluoroethylene (ePTFE) membranes are exposed, germs may seep through and cause illness [34]. Reconstructive surgical techniques in conjunction with implant-plasty may improve peri-implant clinical characteristics, such as pocket-probing depth, pus, and sulcus bleeding, in addition to the longevity of rough-surfaced implants impacted by peri-implantitis [29]. After six months of non-submerged recovery, the study by Schwarz et al. showed that directed bone regeneration and nanocrystalline hydroxyapatite both produced clinically significant improvements in clinical measures [35]. The two treatment modalities were effective in achieving clinically noteworthy decreases in the depth of the probing pocket and a rise in clinical attachment level, as shown by the two-year results of the same clinical study by Schwarz et al. [28]. Nevertheless, the utilization of real bone mineral and collagen membranes appeared to be associated with significant increases in those clinical indicators, implying reliable and improved healing results. Unfortunately, a valid statistical comparison of the effectiveness of the two therapy techniques was not possible due to the study's very small sample size (22 patients). In broad terms, more information has to be gathered on the various regenerative methods used to treat peri-implantitis [36]. According to research by Renvert et al., in shallow peri-implant lesions with a mean probing pocket depth of less than 4 mm, antimicrobial treatment added to mechanical debridement did not offer supplementary advantages [37]. Thus, it appears that in shallow peri-implant lesions with mean pocket probing depth <4 mm, antimicrobial treatment added to mechanical debridement does not offer supplementary advantages. However, in profound peri-implant lesions with mean pocket probing depth >5 mm, it appears to offer further therapeutic benefits. As an outcome, supplemental peri-implant therapies have been proposed to improve non-surgical treatment options for peri-implant mucositis and peri-implantitis. Antibiotics, antiseptics, ultrasonic, and laser treatments are examples of these treatments. Implants positioned in non-esthetic areas are often the only ones subject to surgical excision, and a surgical flap aids in the involved implant's thorough debridement and cleaning [38].

## Conclusions

Comprehensive management is advised for the success of implant treatment. Starting from the patient selection and augmentation procedures from the clinician's side to proper follow-up and oral hygiene maintenance from the patient's side consists of a proper treatment plan. Soft tissue problems must be managed and prevented to avoid negative results in implant dentistry. The clinical features of each case, as well as the patient's wants and demands, determine the treatment's type and timing. Before any soft tissue management surgery, a complete examination of the patient's medical history, periodontal health, bone quality and quantity, and restoration needs should be carried out. Soft or hard tissue quantity and quality should be maintained surgically. Implant site hygiene is mandatory to avoid peri-implant mucositis and peri-implantitis. In case of any complications, a quick intervention is necessary.
